# Low-motion fMRI data can be obtained in pediatric participants undergoing a 60-minute scan protocol

**DOI:** 10.1038/s41598-020-78885-z

**Published:** 2020-12-14

**Authors:** Corey Horien, Scuddy Fontenelle, Kohrissa Joseph, Nicole Powell, Chaela Nutor, Diogo Fortes, Maureen Butler, Kelly Powell, Deanna Macris, Kangjoo Lee, Abigail S. Greene, James C. McPartland, Fred R. Volkmar, Dustin Scheinost, Katarzyna Chawarska, R. Todd Constable

**Affiliations:** 1 Interdepartmental Neuroscience Program, Yale School of Medicine, New Haven, CT USA; 2 MD-PhD Program, Yale School of Medicine, New Haven, CT USA; 3 Yale Child Study Center, New Haven, CT USA; 4 Department of Radiology and Biomedical Imaging, Yale School of Medicine, New Haven, CT USA; 5 Department of Psychology, Yale University, New Haven, CT USA; 6 Department of Statistics and Data Science, Yale University, New Haven, CT USA; 7 Department of Pediatrics, Yale School of Medicine, New Haven, CT USA; 8 Department of Neurosurgery, Yale School of Medicine, New Haven, CT USA; 9 Magnetic Resonance Research Center, 300 Cedar St, PO Box 208043, New Haven, CT 06520-8043 USA

**Keywords:** Functional magnetic resonance imaging, Paediatric research

## Abstract

Performing functional magnetic resonance imaging (fMRI) scans of children can be a difficult task, as participants tend to move while being scanned. Head motion represents a significant confound in fMRI connectivity analyses. One approach to limit motion has been to use shorter MRI protocols, though this reduces the reliability of results. Hence, there is a need to implement methods to achieve high-quality, low-motion data while not sacrificing data quantity. Here we show that by using a mock scan protocol prior to a scan, in conjunction with other in-scan steps (weighted blanket and incentive system), it is possible to achieve low-motion fMRI data in pediatric participants (age range: 7–17 years old) undergoing a 60 min MRI session. We also observe that motion is low during the MRI protocol in a separate replication group of participants, including some with autism spectrum disorder. Collectively, the results indicate it is possible to conduct long scan protocols in difficult-to-scan populations and still achieve high-quality data, thus potentially allowing more reliable fMRI findings.

## Introduction

Functional magnetic resonance imaging (fMRI) has proven to be a powerful tool to study brain function. A promising approach using fMRI data is to measure functional connectivity, in which time courses of the blood oxygen level-dependent (BOLD) signal are correlated across regions of interest^1^. Such analyses have been used extensively to characterize the brains of younger children^2–6^, with the aim of one day potentially being useful in clinical settings^7–9^.

Nevertheless, scanning children can be challenging, especially because younger children tend to move while being scanned. The effect of motion on measures of functional connectivity is well documented^10,11^, and it can introduce major confounds into analysis pipelines. While there are post-hoc methods to clean fMRI data to reduce the impact of motion^12–17^, there is no consensus about the best way to do so^18,19^.

One approach to decrease in-scanner motion is to limit it during data acquisition. A common strategy with children is to use shortened scan protocols and prioritize the scans of interest—for example, collecting only a structural scan for common-space template registration and only 1–2 resting state or task-based scans. However, numerous groups have shown that the reliability of functional connectivity measures decreases when using fewer scans or scans of a shorter duration^20–24^. Thus, investigators choosing a shortened scan protocol run the risk of obtaining unreproducible results.

In addition, other in-scanner methods exist to help obtain low-motion data. One approach, Framewise Integrated Real-time MRI Monitoring (FIRMM), analyzes head motion in real-time and allows the scanner operator to collect data until a satisfactory amount of low-motion data have been obtained^25^. Other groups have demonstrated that showing movies—actual movie clips^26^ and clips of abstract shapes (e.g., *Inscapes*^27^)—during a scan can reduce motion in younger children. While these approaches are helpful, there are potential issues with each. For instance, it would be difficult to use FIRMM when completing task-based scans, as differences in task length might affect task performance and confound analyses. Showing participants movie clips affects connectivity, even when using a low-demand movie such as *Inscapes* with minimal semantic content^26,27^. Because of the impact on connectivity, it would be preferable to decrease motion without introducing confounds.

Mock scanning protocols are one potential solution to the problem of in-scanner motion. While exact details vary, this approach typically entails placing participants in an environment designed to mimic the real scanning environment, desensitizing them, and training them to limit movement. Numerous groups have described successful implementation of a mock scan protocol and have shown that it can be used to limit in-scanner motion in younger children^28–32^. However, these studies have tended to be shorter in duration (i.e. between 20 and 40 min to scan one participant) and have tended to collect only structural and/or a few task or resting-state scans, so the efficacy of mock scanning when using longer MRI protocols is unclear. In addition, most of these studies have lacked a control group, rendering effectiveness of the mock scan unquantifiable.

Our aim in this paper is three-fold: (1) build on the tradition of using mock scans and show that such an approach can be used with in-scan methods to achieve low-motion data in pediatric participants (age range: 7–17 years old) undergoing a 60 min MRI protocol; (2) compare results to a group who did not receive a mock scan or the other in-scan steps; and (3) assess generalizability of the protocol by having a separate group of researchers conduct the mock scans and the in-scan steps and then quantify motion in this group. In sum, the results indicate it is possible to achieve low-motion fMRI data when performing long MRI protocols in children, and preliminary results suggest that such low-motion data can be achieved in participants with autism spectrum disorder (ASD). We also describe our protocol in detail and offer practical tips for other researchers wishing to implement our methods.

## Results

### High- and low-motion scans in the informal and formal mock scan groups

We first set out to determine if it was possible to obtain low-motion functional data in a group of participants undergoing a mock scan protocol (see Supplemental Methods, Supplemental Figs. S1, S2 and Supplemental Table S1 for a full description of the protocol) and in-scan steps (weighted blanket and in-scan incentive system; see “Methods”). We are not interested in the individual effect of the mock scan compared to the blanket or the in-scan incentive system in limiting motion in this group of participants (hereafter the “formal mock scan group”). Rather, we are interested in whether all three, used together, can limit movement relative to a group of participants who did not undergo the mock scan or in-scan steps (hereafter the “informal mock scan group”). We note the data presented here were collected as part of an ongoing project to study functional connectivity alterations in ASD, and participants were assigned to a group based on when they enrolled in the study (see “Methods”). That is, subject assignment was not random, and the associational nature of this study must be kept in mind when interpreting comparisons between the groups (see “Limitations” in “Discussion”).

The demographic characteristics of these groups were not significantly different in terms of age, sex, or measures of IQ, and none of the participants in either group had ASD (Table 1). To quantify motion, we calculated mean frame-to-frame displacement (FFD) over a functional scan, which gives a scalar value in millimeters of how much the participant moved over each time point. Eight functional scans were conducted over the course of a 60 min MRI session: two runs of an attention task (referred to as ‘gradCPT’ below), four runs of movie clips, and two resting-state runs (see Supplemental Methods for a description of the functional runs). With task and eye-tracker setup, total time spent in the magnet was typically 80–90 min (Supplemental Table S1).

**Table 1:** Demographic information.

Measure: group means (s.d.)	Informal mock scan group	Formal mock scan group	Replication group	*P*-value (formal mock scan group vs. informal mock scan group)	*P*-value (replication group vs. informal mock scan group)
Number of participants	7	12	16	–	–
Number of participants with ASD	0	0	5	–	–
Age in years	10.24 (2.05)	11.31 (2.78)	12.14 (2.89)	0.39	0.13
Males per group	6	6	5	0.32	0.03
IQ: verbal	118.28 (10.81)	117.5 (10.74)	114.06 (12.22)	0.88	0.44
IQ: non-verbal	116.43 (13.25)	108.9 (10.72)	108.81 (12.01)	0.23	0.19
IQ	119 (15.44)	113.08 (12.9)	111.56 (11.25)	0.38	0.21

**Figure 1:**
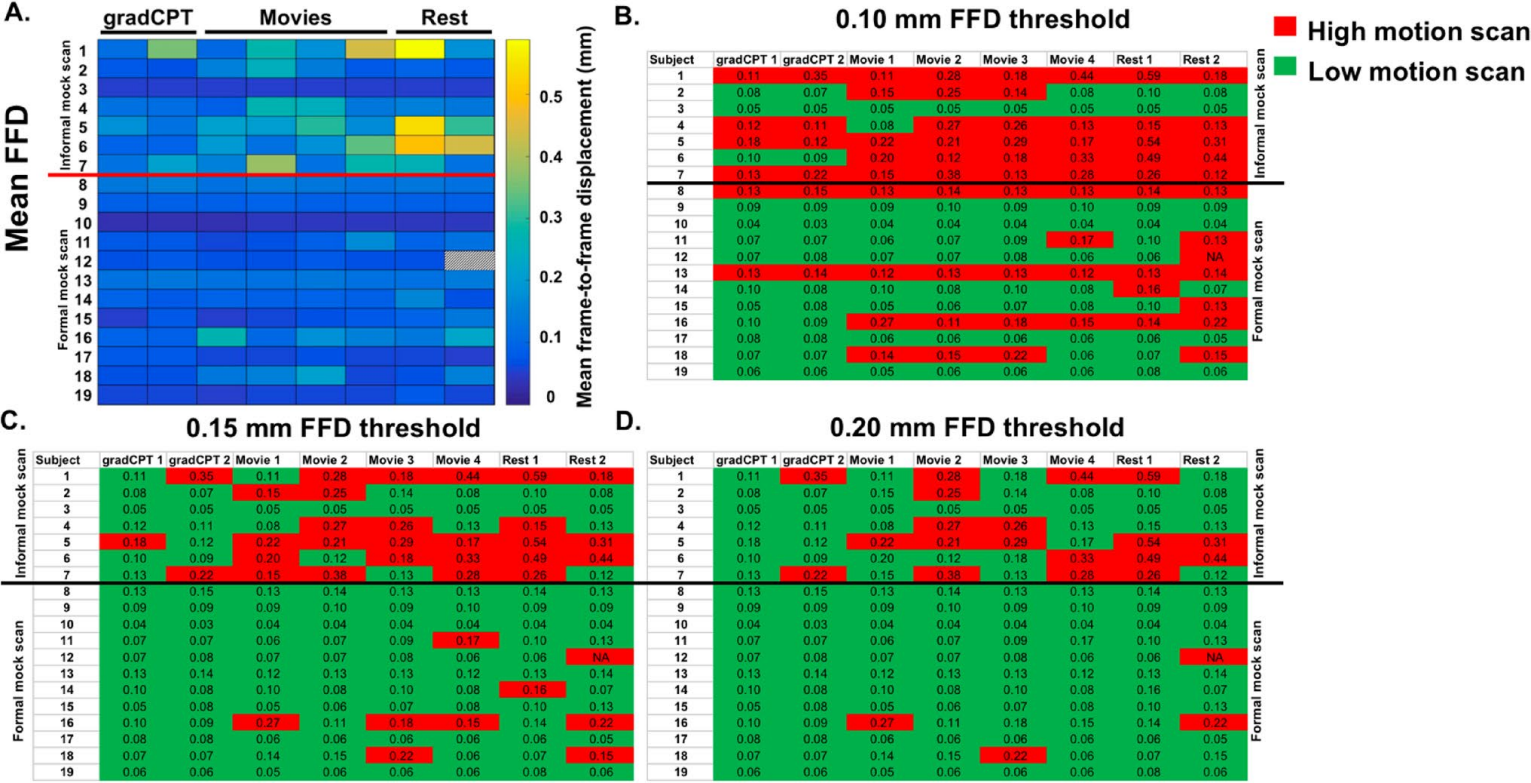
Low-motion data can be obtained in the formal mock scan group. For all plots (**a**–**d**), each row represents a different participant; each column indicates a different scan condition (scan labels are shown at the top of each plot). Participants in the informal mock scan group are shown in the first 7 rows; those in the formal mock scan group are shown in rows 8–19. The line separating participant 7 and 8 divides participants into their respective groups; group labels are shown on the side of each plot. Note that the data are presented in functional run order: the gradCPT scans are the first functional scans conducted; the rest scans are the last functional runs conducted. (**a**) A matrix of the mean FFD values for each participant. (**b**–**d**) The same data from (**a**) are plotted, except scans are classified as being either a “high-motion” scan (i.e. the scan had a mean FFD above the relevant threshold) or a “low-motion” scan (the scan had a mean FFD below the threshold). The mean FFD value for each scan is shown in each cell. Note that for visualization, we have rounded the mean FFD value to two significant figures; when we classified a scan as low or high-motion, we used four significant figures. Also note that in all figures, subject 12 (in the formal mock scan group) did not complete the last rest scan. In (**a**), this scan is shown with grey hatched lines; in (**b**–**d**), we considered this a high-motion scan (*FFD* frame-to-frame displacement; *mm* millimeters).

To give a sense of the distribution of mean FFD values of these two groups, we show the mean FFD of each scan through multiple visualizations. First, we show the data in matrix format, in which each cell is colored according to the mean FFD of the participant for that scan (Fig. 1a). Visual inspection reveals most of the high mean FFD scans belong to participants in the informal mock scan group. When mean FFD thresholds are imposed, a common procedure to limit the impact of motion on functional connectivity analyses^33–37^, a similar trend emerges (Fig. 1b–d). For example, at a threshold of 0.10 mm, 71.4% of the scans from the informal mock scan group are high-motion (i.e. have mean FFD above 0.10 mm); only 32.3% of the scans from the formal mock scan group are high-motion. The difference in high-motion scans was statistically significant (Pearson Chi-square = 21.76, *P* < 0.001). We obtained similar results when using thresholds of 0.15 mm (50% of the scans from the informal mock scan group are high-motion; only 9.38% of the scans from the formal mock scan group are high-motion; Pearson Chi-square = 31.69, *P* < 0.001) and 0.20 mm (33.9% and 4.17% of the scans are high-motion in the informal mock scan and the formal mock scan groups, respectively; Pearson Chi-square = 24.4, *P* < 0.001).

### Comparison of mean FFD values between informal and formal mock scan groups

**Figure 2:**
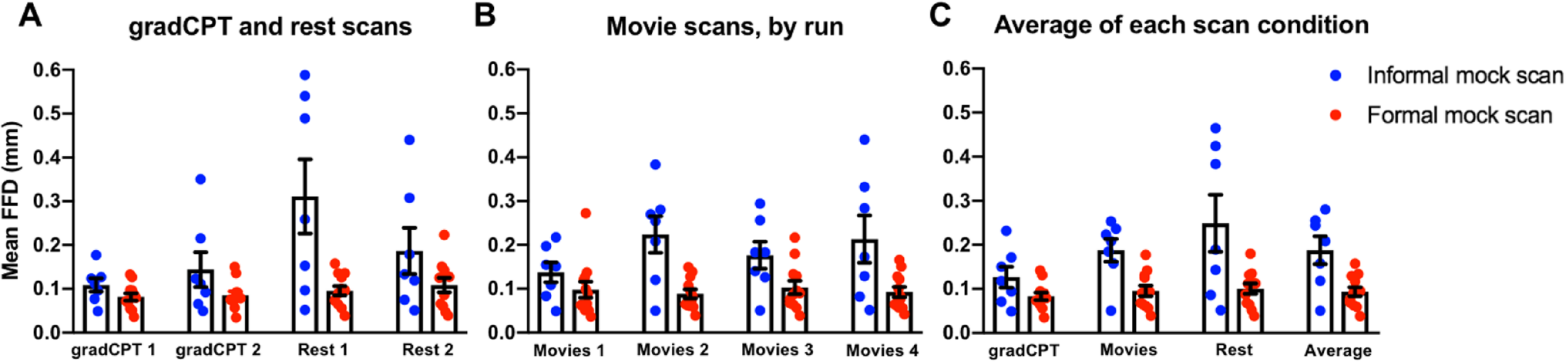
Low-motion data can be obtained in the formal mock scan group across different scan conditions. For all plots (**a**–**c**), the scan condition is shown below the x-axis; the mean FFD (mm) is shown on the y-axis. The average mean FFD for each group/condition is shown as a bar; error bars correspond to standard error of the mean. (**a**): mean FFD values for gradCPT and rest scans. (**b**): mean FFD values for the movie scans. (**c**): the average mean FFD value for each condition. The grand mean FFD over all eight functional scans is shown to the right of the plot and is referred to as “Average” under the x-axis (*FFD* frame-to-frame displacement; *mm* millimeters).

We next assessed motion across the different scan conditions. Rather than classifying a scan as either high- or low-motion, we analyzed the mean FFD values for each participant (Fig. 2). We found that across the different task and rest runs, the formal mock scan group tended to have lower mean FFD values relative to the informal mock scan group. Across all conditions, the effect size tended to be large (Table 2; see “Methods” for a discussion about effect size interpretation). The differences in group movement were significant across all conditions tested (except gradCPT; Table 3), such that the formal mock scan group had a statistically lower mean FFD group average compared to the informal mock scan group. In addition, we observed that when grouping the movie data into clips of interest (Supplemental Table S2; Supplemental Fig. S3), the formal mock scan group again had lower motion scans than the informal mock scan group. To ensure the higher number of males in the informal mock scan group was not driving the difference in motion, we restricted the comparison to only males; we observed the formal mock scan group still had lower motion data (Supplemental Table S3).

**Table 2:** The effect size of the difference between the average mean FFD value of the informal and formal mock scan groups.

Condition	Hedge’s *g*	Effect size^a^
gradCPT 1	0.82	Large
gradCPT 2	0.87	Large
Movie run 1	0.63	Medium
Movie run 2	1.89	Large
Movie run 3	1.14	Large
Movie run 4	1.33	Large
Rest 1	1.58	Large
Rest 2	0.81	Large
Average gradCPT	0.82	Large
Average movie	1.26	Large
Average rest	1.21	Large
Average over all conditions	1.08	Large

^a^We used the following criteria for effect size interpretation: small (g ≥ 0.2), medium (g ≥ 0.5), and large (g ≥ 0.8)^38^.

**Table 3:** Statistical significance of the difference between the grand mean FFD value of the informal and formal mock scan groups.

Condition	Degrees of freedom	*t*-statistic	*P*-value (FDR corrected)	Formal mock scan group mean FFD significantly lower than informal mock scan group?
gradCPT	17	2.05	0.0561	No
Rest	17	2.93	0.0094	Yes
Movies	17	3.7	0.0018	Yes
Average across all scans	17	3.48	0.0029	Yes

### Results in replication group

Because the same individual (CH) led the mock scans and the in-scan steps, it is possible that the results we obtained were specific to this individual. To control for this potential confound, three other authors were trained to conduct the mock scans and administer the in-scan incentive system. Motion was then assessed in this separate group of subjects (hereafter referred to as the “replication group”). Of note, we included 5 participants with ASD in the replication group, to assess if we were able to obtain low-motion data in this typically difficult-to-scan population. Demographic characteristics of this group were similar to the informal mock scan group in terms of age, IQ, and sex, after correcting for multiple comparisons (Table 1).

**Figure 3:**
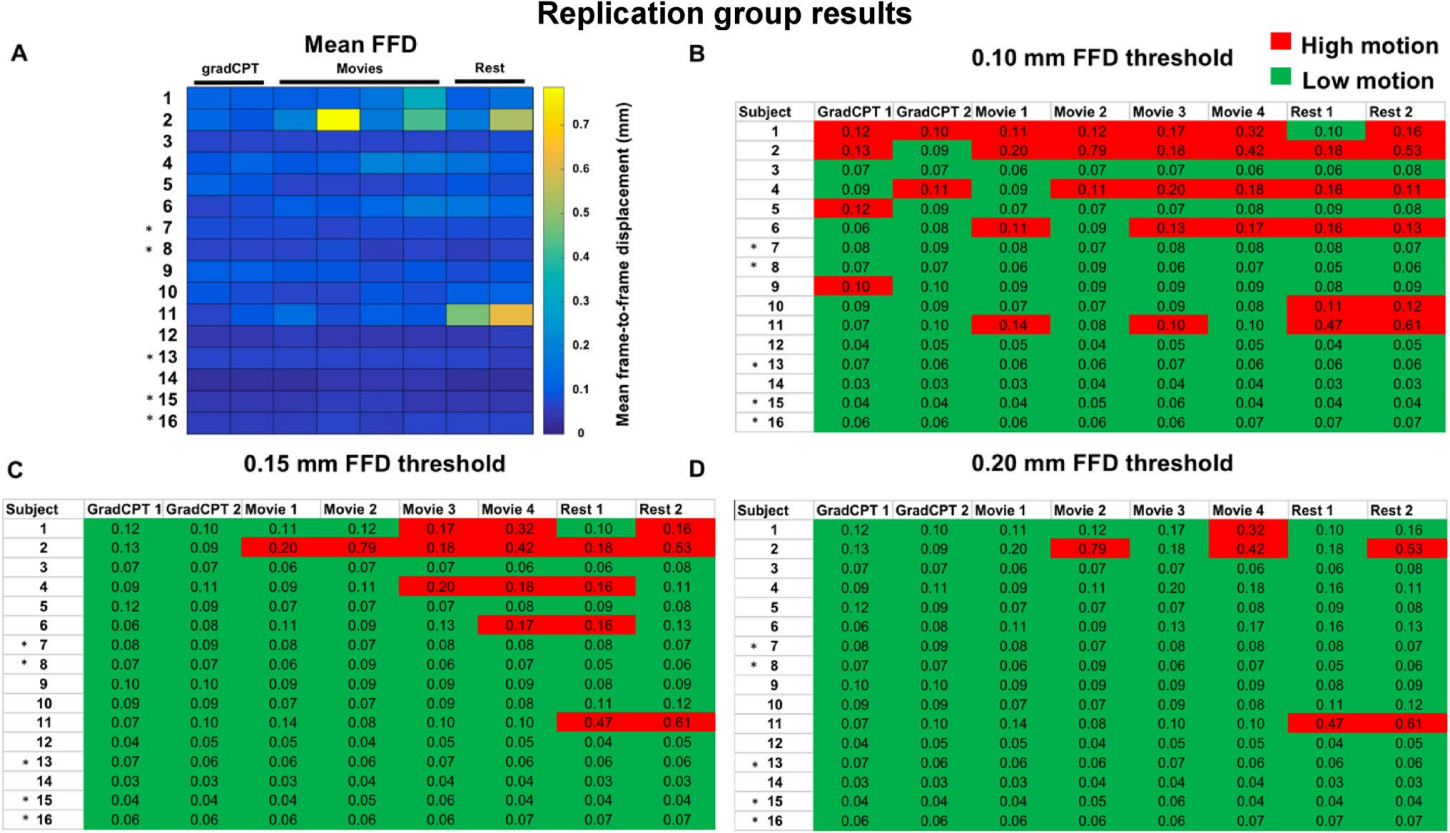
Low-motion data can be obtained in a replication group of participants. Participants with ASD are denoted by an asterisk (‘*’) to the left of subject number in each plot. For all plots (**a**–**d**), each row represents a different participant; each column indicates a different scan condition (scan labels are shown at the top of each plot). Note that the data are presented in functional run order: the gradCPT scans are the first functional scans conducted; the rest scans are the last functional runs conducted. (**a**) A matrix of the mean FFD values for each participant. (**b**–**d**) The same data from (**a**) are plotted, except scans are classified as being either a “high-motion” scan (i.e. the scan had a mean FFD above the relevant threshold) or a “low-motion” scan (the scan had a mean FFD below the threshold). The mean FFD value for each scan is shown in each cell. Note that for visualization, we have rounded the mean FFD value to two significant figures; when we classified a scan as low or high-motion, we used four significant figures (*FFD* frame-to-frame displacement; *mm* millimeters).

Similar to the formal mock scan group, we were able to obtain low-motion scans in the replication group (Fig. 3a). At the 0.10 mm, 0.15 mm, and 0.20 mm FFD thresholds, we observed similar proportions of low-motion to high-motion scans as in the formal mock scan group (Fig. 3b–d). Of note, all participants with ASD had low-motion scans at each threshold. When we compared the mean FFD values obtained from a scan condition, as well as the average across all scans within a participant, we again found that the replication group exhibited a statistically significant lower mean FFD compared to the informal mock scan group (except for the rest condition; Table 4). Calculation of effect sizes revealed that the difference in mean FFD relative to the informal mock scan group was large for all conditions, as well as the average of all scans (Table 4; see “Methods” for a discussion about effect size interpretation). When we restricted the comparison to only males, we observed the replication group again had lower motion data (Supplemental Table S3).

**Table 4:** Effect size and significance testing of replication group compared to the informal mock scan group.

Condition	Degrees of freedom	*t*-statistic	*P*-value (FDR corrected)	Replication group mean FFD significantly lower than informal mock scan group?	Hedge’s *g*	Effect Size
gradCPT	21	2.77	0.0115	Yes	1.25	Large
Rest	21	1.83	0.0815	No	0.83	Large
Movies	21	2.27	0.0339	Yes	1.03	Large
Average across all scans	21	2.49	0.0212	Yes	1.13	Large

## Discussion

We have described steps our group has taken to limit in-scanner motion in pediatric participants undergoing a 60-min fMRI protocol. By implementing the mock scan protocol described here, in addition to in-scan steps, we have significantly limited movement in this sample. These findings were robust to experimenter, as demonstrated by the low-motion data we obtained in two separate groups of participants. Taken together, these preliminary results indicate that our approach can be used to limit motion in pediatric participants undergoing a long MRI protocol.

### Extending the mock-scan protocol literature by conducting longer MRI protocols

Scanning younger participants is a difficult task, especially those with a clinical condition or neurodevelopmental disorder. The use of mock scanning protocols to increase MRI scan quality has been well documented^28,29,31,32,39^. Nevertheless, these studies have tended to use shorter MRI scan protocols (i.e. only 20–40 min) and to acquire only structural and a few resting-state scans. We extended this work by showing the efficacy of a mock scan protocol in an MRI study that is longer (e.g. 60 min of total scan time; 80–90 min in the scanner) and uses a variety of task-based as well as rest scans. We make no claim about the novelty of our mock scan protocol or the in-scan steps. Our aim is simply to report that using such methods can be used to achieve high-quality data during a long MRI scan session. The fact that we were able to perform a long MRI protocol and achieve comparable rates of low-motion data to other studies using mock scans is encouraging^28,30,32,39^, given that reliability of functional connectivity increases with more data per subject^20–24^. Hence, our protocol paves the way for potentially more reproducible fMRI results.

### Comparison with other methods to reduce in-scanner motion

Given the effect of motion on functional connectivity analyses^10,11^, acquiring high-quality data with minimal motion is of the utmost importance. As such, many groups have implemented steps before and during scans to limit participant movement. While these methods have proven useful, they are often not flexible enough to be applicable to all functional scans in a protocol (i.e. FIRMM can only be used in rest scans^25^), or they require researchers to provide feedback during a task-based scan, which might not be desirable for a given task^26^. The use of sedation or anesthesia to increase compliance has also been described^40–42^, but it is well-acknowledged that such an approach can also affect connectivity^43–45^. The methods we have documented here circumvent these issues: they result in acquisitions with lower motion across both task and rest scans, do not require the use of additional stimuli during scans, and do not require sedation.

### Other motion considerations

We have described here one approach to limit motion. Future work may seek to combine our methods with other ways of reducing in-scanner motion. For example, one could use our mock-scan protocol before the MRI session. In the MRI session, a resource like FIRMM^25^ could be used during the resting-state scans, specific feedback during or in between scans could be provided to participants^26^, and/or resources like *Inscapes*^27^ could be utilized as desired.

We also point out two other pertinent ways to decrease in-scanner motion that could be used in conjunction with a mock scan. The use of bite-bars is well known to many in the fMRI field. However, many participants describe bite bars as being extremely uncomfortable, and they have not been widely adapted by fMRI research groups. Custom head molds are another attractive option for reducing head-motion^46^. Nevertheless, it is unclear how younger participants, especially those with a disorder, would respond to such a head mold, as the work demonstrating the efficacy of this approach was conducted in healthy subjects that were older than the majority of subjects tested here.

### Use with participants with ASD

We found that we were able to obtain low-motion data in all participants who had ASD. Though the small number of subjects limits strong conclusions, these initial results are encouraging. Other groups have documented a number of methods to prepare participants with ASD for MRI scans, including mock scan protocols^30,47,48^, as well as motivational techniques and individualized prize systems^30^. Our results add to this literature and provide evidence that such tools can be utilized to perform longer MRI protocols in this typically difficult-to-scan population. Using such tools is important, because we have known since the National Research Council report on autism^49^ that some children with ASD are unresponsive to treatment, and the ability to include these children in longer neuroimaging studies may help us clarify differences in brain function that contribute to a lack of treatment response.

### Limitations

The small sample size of all three groups is a primary limitation, especially of the informal mock scan group. Put simply, the small samples are not ideal for calculating robust estimates of the effect of the protocol. It is likely effect sizes are somewhat inflated and that larger studies, with more participants, might not observe similar rates of low-motion data. However, given that we achieved low-motion data in two separate groups (i.e. the formal mock scan and replication groups), this increases confidence in the efficacy of the mock scan protocol and in-scan methods. Another limitation is that because the sample described here is part of a larger, ongoing study, subject assignment was not random. It is possible the nonrandom subject assignment into study groups biased effect sizes. In the future, randomized studies could be conducted with larger samples, permitting a more causal relationship to be observed between the protocol and in-scan motion. In addition, the three study groups had IQs that were higher than average. The methods described here may only work in individuals with a high IQ, and results might not generalize to other groups. Our data must be interpreted in the context of this potential confound, and our mock scan and in-scan methods might have to be adapted to fit the needs of other populations.

Another important confound is the unequal number of males and females in each group. By comparing motion estimates from the formal mock scan and replication groups (composed of approximately equal male and female participants) to the informal mock scan group (which was composed mostly of male participants), it is possible such a comparison is confounded by the higher number of males in the informal mock scan group. When we restricted analyses to only males to control for this confound, we observed lower motion data in the formal mock scan and replication groups, which was in line with results when the entire groups were compared. Nevertheless, the sex imbalance among our groups is an unfortunate reality of the sample and a consequence of deriving these data from an ongoing study, in which evaluating efficacy of the mock scan protocol is not the primary goal. Hence, we point out the sex imbalance among the groups to allow readers to transparently evaluate our results.

Two final considerations warrant discussion. Participants in the formal mock scan and replication groups received a mock scan that was much longer (~ 45 min longer) than the informal mock scan group. The much longer duration of the protocol, along with the increased social contact with research staff, might be playing a role in the data observed here, and these caveats must be kept in mind. Future studies could determine the effect of time and social interaction on the efficacy of mock scan protocols. Indeed, having participants complete a formalized mock scan prior to their actual scan might not be feasible due to scheduling and cost constraints. More work could be conducted to determine the effectiveness of a more streamlined mock scan protocol.

## Conclusion

In summary, our data indicate it is possible to conduct long scan protocols in difficult-to-scan populations through the use of a mock scan protocol and in-scan methods. Importantly, high quality data can still be achieved, thus potentially allowing more reliable fMRI findings.

## Methods

To limit the space of this section, we refer the reader extensively to the Supplemental Methods, where we offer full descriptions of the mock scan environment, the mock scan protocol, and tips for other investigators wishing to implement our methods.

### Participants

The present sample was derived from an ongoing study being conducted to study functional connectivity alterations in ASD. This study has been approved by the Yale University Institutional Review Board, and all work described here was performed in accordance with the relevant guidelines and regulations. Informed consent was obtained from the parents of participants. Written assent was obtained from children ages 13–17; verbal assent was obtained for participants under the age of 13. All participants and families provided informed consent for publication of participant data (including potentially identifying information/images) in an online, open-access publication. As appropriate, we have adhered to the Strengthening the Reporting of Observational Studies in Epidemiology (STROBE) guidelines for reporting results related to observational studies^50^. Participants were screened over the phone for basic developmental history and MRI safety factors. Those with a history of prematurity, known genetic abnormalities, or an IQ below 70 were excluded. Diagnostic classification of ASD was based on clinical best estimate diagnosis by a team of clinical psychologists and any available reports of developmental and medical history (see Table 1 for demographic information).

### Study groups

Participants were assigned into a study group based on when they enrolled in the study. The first group of participants received a 5–10 min informal mock scan (n = 7; none with ASD) and served as the comparison group for this study. (See “Supplemental Methods” for a description of the informal mock scan.) In addition, this group did not undergo the in-scan steps—they did not use a weighted blanket during the scan and did not partake in the in-scan incentive system (described below). No participant dropped out before or during the scan. We refer to this group in the main text as the “informal mock scan group”.

The second group of participants (n = 14; none with ASD) took part in a formal mock scan with intensive feedback (Supplemental Methods; Supplemental Figs. S1, S2; Supplemental Table S1) and underwent the pre-scan steps to limit motion. Two of the 14 (14%) participants were unable to complete the mock scan due to expressed discomfort and were excluded. The final sample size of this group was n = 12. The corresponding author (CH) conducted the mock scans and in-scan steps. We refer to this group in the main text as the “formal mock scan group”.

The third group of participants received a formal mock scan (n = 16; 5 with ASD) with intensive feedback led by two other members of the research staff other than CH (NP, KJ, CN, DF, MB) and underwent the in-scan steps. The purpose of this group was two-fold: first, to determine if low-motion data could be obtained in a group of participants who had not been primarily trained by CH, and second, to determine if the protocol could be used in individuals with ASD. We refer to this group as the “replication group”.

### MRI session

After a localizer, participants underwent the following scans (Image acquisition parameters described in Supplemental Methods): anatomical magnetization prepared rapid gradient echo (MPRAGE); T1 fast low angle shot (T1 FLASH); two runs of an attention task (the gradual onset continuous performance task; gradCPT^51–53^); T2-weighted 3D fast spin echo image (T2 SPACE); four movie runs^54,55^, in which participants were shown clips of an actress with and without eye-contact, with and without speech (Supplemental Methods; Supplemental Fig. S4); and two resting-state runs. The total scan duration was 61 min, 13 secs. Eye-tracking data are being collected in this study, adding approximately 5–10 min for participant setup and 5–10 min for calibrating and validating the eye-tracker before data collection. Total time spent in the magnet ranges from 80 to 90 min (Supplemental Table S4).

### In-scan steps to limit motion

In addition to the mock scan protocol, we took other measures to limit motion in the formal mock scan group and replication group. We used an MRI-compatible weighted blanket during the actual MRI session (https://www.mosaicweightedblankets.com/). We customized the blanket so it would be appropriately sized for children (blanket dimensions: 38″ × 60″; eight pounds of total weight).

We also implemented a prize system during the actual MRI session for both the formal mock scan and replication groups. Participants could win up to three prizes (toys, stickers, etc.) of their choice for low-motion scans. A participant could win their first prize if they stayed still over both gradCPT functional runs, their second if they stayed still over the movie runs, and their third if they stayed still over the rest scans. A “low-motion scan” was determined based on watching the participant during the scan through the eye-tracking camera and also by determining if the subject moved between frames by manual inspection. We intentionally allowed for the prize system to be flexible. If it was deemed that a younger subject would respond positively to prizes, we would increase the number of prizes they could win up to five. If an older subject was not interested in prizes, we did not force them to pick out prizes before the scan. In our experience, this flexibility can help increase the number of usable scans.

### Quantification of motion

To quantify subject motion, we calculated the mean frame-to-frame displacement (FFD) of the patient’s head across each functional run. We estimated a set of motion parameters using SPM8^56^ to obtain a transformation matrix $T$ at frame $i$ that allowed us to map the average Euclidean distance the center of gravity (${\text{COG}}$) of each frame moved. We then calculated the average movement over all frames acquired during a functional run. In equation form, we computed

$${\text{mean}}\,FFD= \frac{1}{n} \sum \sqrt{{{(D}_{x}\left(i\right)-{D}_{x}\left(i+1\right))}^{2 }+{{(D}_{y}\left(i\right)-{D}_{y}\left(i+1\right))}^{2}+{{(D}_{z}\left(i\right)-{D}_{z}\left(i+1\right))}^{2}}$$

where $n$ = number of frames, $i$ = time point, and ${D}_{x}\left(i\right)$, ${D}_{y}\left(i\right)$, and ${D}_{z}\left(i\right)$ are the x, y, z elements of $T\left(i\right)*{\text{COG}}$ at time $i$, respectively, leaving us with a scalar estimate of motion (mean FFD in mm).

### Differences in high- and low-motion scans

If a participant’s scan had a mean FFD above a threshold, it was designated as a “high-motion” scan; if it was below the threshold, it was considered “low-motion.” Mean FFD thresholds of 0.10, 0.15, and 0.20 mm were used, as thresholds of this magnitude have been shown to limit the impact of motion^21,33^ while allowing for sample sizes of adequate size in children/adolescents^33,34^ and in those with a disorder^35,36,57^. We used a Chi-square test of association to determine if there was a significant difference in the number of high and low-motion scans; significance was assessed at a *P*-value < 0.05 after correcting for multiple comparisons (Benjamini–Hochberg procedure^58^).

### Differences in mean FFD

To determine if the mean FFD values differed due to scan condition (movies, gradCPT, and rest), we calculated Hedge’s *g*^59^, the preferred estimate of effect size when sample sizes are small^60^ and unequal^61^. We used the criteria suggested by Sullivan and Feinn^38^ for effect size interpretation. We present effect sizes here simply as converging evidence—the associational nature of this study and the nonrandom subject assignment into groups must be kept in mind when interpreting the magnitude of effect size estimates. To determine statistical significance, we performed two-sample *t*-tests on the average mean FFD value for a condition (i.e. for the rest condition, we averaged the mean FFD value from both rest scans for a participant). Significance was assessed at a *P*-value < 0.05 and corrected for multiple comparisons as above. We note the same caveats affecting effect size interpretation apply to the results of statistical testing.

## Supplementary Information

Supplementary Information.
