# Supplementary material for: Low-motion fMRI data can be obtained in pediatric participants undergoing a 60-minute scan protocol
Source: Sci Rep. 2020 Dec 14;10:21855. doi: 10.1038/s41598-020-78885-z (PMC7736342; doi:10.1038/s41598-020-78885-z)
Supplement: Supplementary file 1 — Supplementary Information. [file 41598_2020_78885_MOESM1_ESM.docx]

**Authors:**

Corey Horien^1,2^*, Scuddy Fontenelle IV^3^, Kohrissa Joseph^3^, Nicole Powell^3^, Chaela Nutor^3^, Diogo Fortes^3^, Maureen Butler^3^, Kelly Powell^3^, Deanna Macris^3^, Kangjoo Lee^4^, Abigail S. Greene^1,2^, James C. McPartland^3,5^, Fred R. Volkmar^3,5^, Dustin Scheinost^1,3,4,6^, Katarzyna Chawarska^3,6,7^, R. Todd Constable^1,4,8^

**Title:**

Low-motion fMRI data can be obtained in pediatric participants undergoing a 60-minute scan protocol

**Affiliations:**

^1^Interdepartmental Neuroscience Program, Yale School of Medicine, New Haven, CT, USA

^2^MD-PhD Program, Yale School of Medicine, New Haven, CT, USA

^3^Yale Child Study Center, New Haven, CT, USA

^4^Department of Radiology and Biomedical Imaging, Yale School of Medicine, New Haven, CT, USA

^5^Department of Psychology, Yale University, New Haven, CT, USA

^6^Department of Statistics and Data Science, Yale University, New Haven, CT, USA

^7^Department of Pediatrics, Yale School of Medicine, New Haven, CT, USA

^8^Department of Neurosurgery, Yale School of Medicine, New Haven, CT, USA

*Corresponding author:

Corey Horien

Magnetic Resonance Research Center

300 Cedar St

PO Box 208043

New Haven, CT 06520-8043

[corey.horien@yale.edu](mailto:corey.horien@yale.edu)

**Supplemental Methods and Results**

Description of mock-scanning environment

The mock scanner consisted of a 5-foot-long motorized bed, a wooden mock head coil, and an audiovisual presentation system (Supplemental Fig. 1). Speakers placed in corners of the room were used to play gradient sounds. To mimic the scanning environment as closely as possible, participants were given earplugs to wear; auditory stimuli were delivered through headphones. A fan inside the bore was also turned on to replicate the scanning environment. Visual stimuli were seen through the use of a mirror system that displayed visual stimuli situated at the back of the bore. MoTrak (<https://pstnet.com/products/motrak/>) was used as the head motion tracking system in this study (Supplemental Fig. 2); this system allows participants to see how their head moves in real-time while they are in the mock scanner. Head movement was monitored through the use of a MoTrak headband. In general, two staff members conducted a mock scan: one interacted with the child and provided feedback; the other staff member set up and monitored the MoTrak software. The staff member monitoring the MoTrak software was able to view the participant through a camera to ensure participant compliance and observe how the participant responded to feedback.


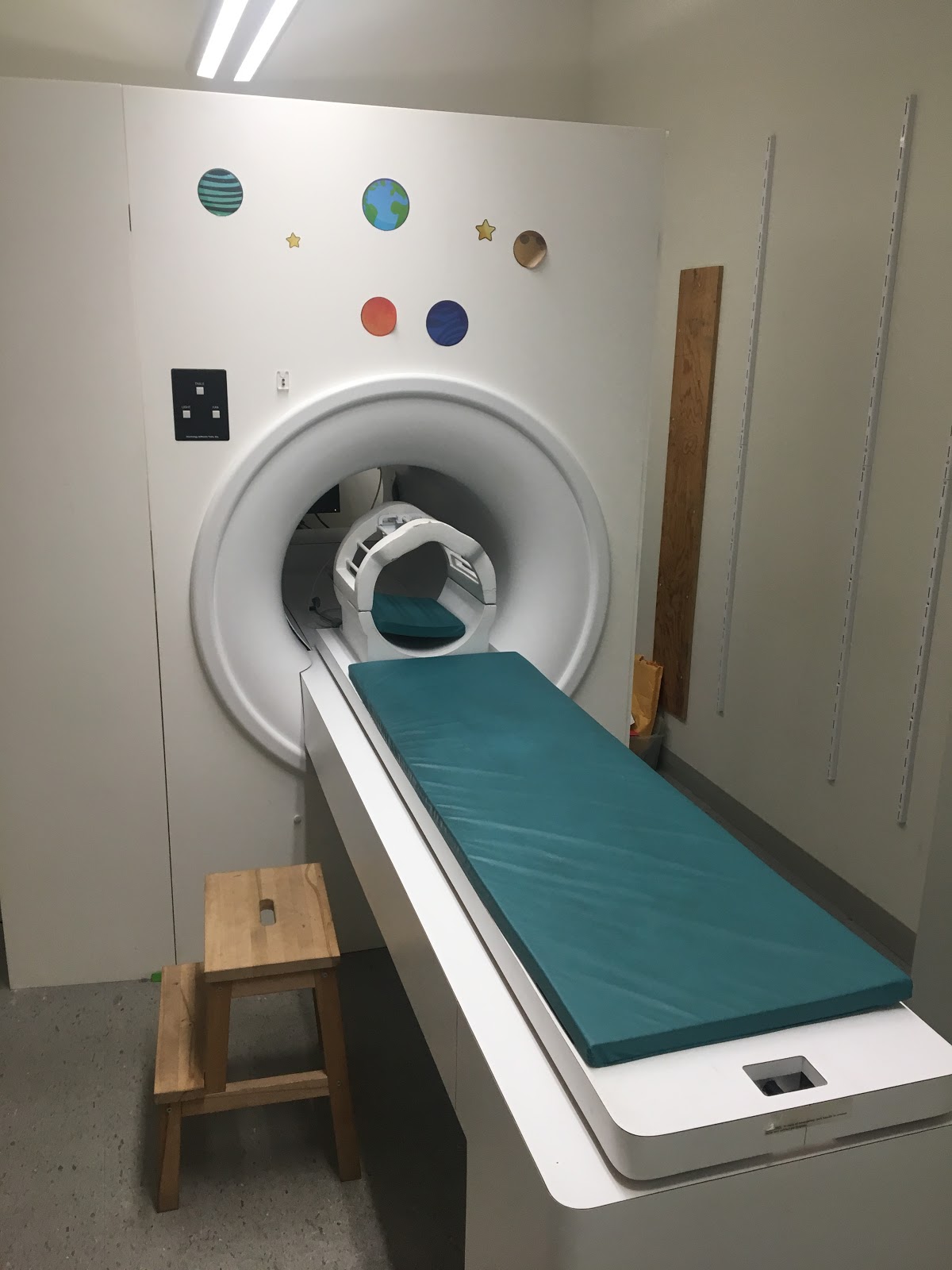


Supplemental Figure 1. The mock-scanner used in this study. A motorized bed is used to move the participant back into the bore. The participant views visual stimuli through the use of a mirror on the head-coil. The participant wears headphones to hear sounds from the movie. Speakers (not visible in the photo) are used to play gradient sounds while the participant lies in the mock-scanner.


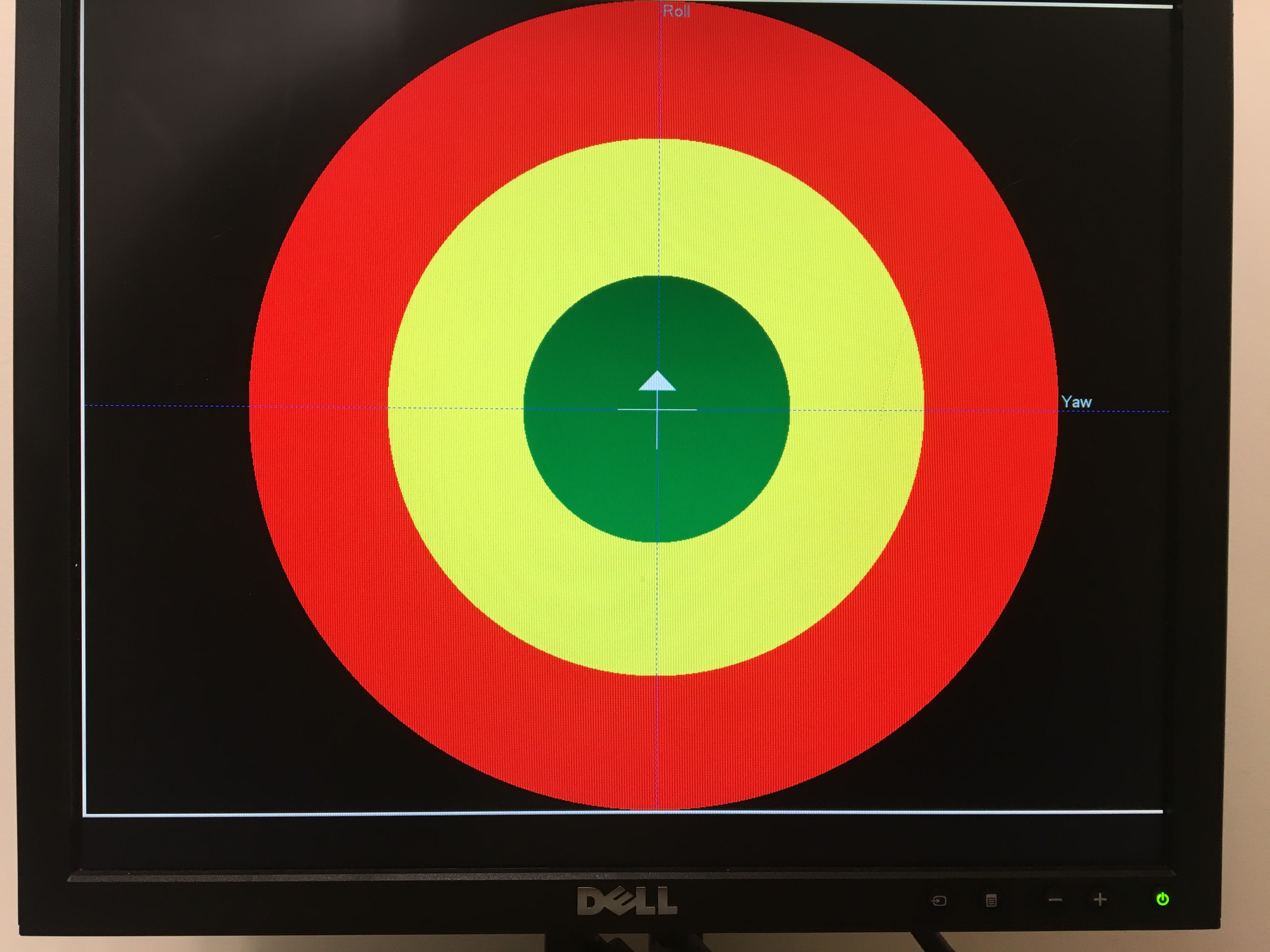


Supplemental Figure 2. The target system used in conjunction with MoTrak software. The cursor (shown in the center of the target as a cross) tracks participant head movement. This screen is visible to the participant at the back of the mock scanner through a mirror system. Head movement results in the cursor moving on the screen—hence, the participant can see how their movement causes the cursor to move. Participants are encouraged to stay in the green circle as much as possible. Depending on the step of the mock scan protocol (see below for more details), verbal feedback is also given to the participant (“You moved your arms, and it caused your head to move; did you see that on the screen?”).

Description of formal mock scan protocol

The mock scan protocol consists of nine steps, including several mock scan runs with incremental incorporation of visual stimuli, scanner sounds, and feedback about motion. The entire sequence takes about 45-50 minutes to complete, and was typically conducted four days before the actual MRI protocol, depending on participant availability. Note that participants in the formal mock scan and replication groups underwent this protocol.

The first step of the mock scan involved introducing the participant to the mock scan environment and was three minutes long (Supplemental Table 1). The participant was allowed to walk around the room, look inside the mock scanner, and ask any questions. Age-appropriate language was used to describe the mock scanner (e.g., for younger participants: “This is a camera we use to take pictures of your brain.”). The goals of the mock scanning session were explained to the participant. (“It is important that when you are in the scanner, you stay very still. We are going to practice that over the next 45 minutes.”) As appropriate, a question and answer approach was utilized by the research staff with the participant: “What happens if you move around a lot when your picture is being taken? That’s right; it is blurry. That is why we need you to stay still when we are taking pictures of your brain.”

The second step included preparing the participant for the mock scan. Ear plugs were given to the participant; after inserting them into the ears, headphones were applied over the ears by research staff, again with the intention of simulating the actual scanning environment as closely as possible. The MoTrak head-tracking headband was applied. The participant then laid back, and the mirror was adjusted so the participant could view the computer monitor placed at the back of the mock scanner. As appropriate, visual stimuli (including the target system from MoTrak, movie clips, or a fixation cross) were projected through the back computer monitor.

After this, the participant lay down in the bore of the mock scanner for three minutes. No visual stimuli were presented. No scanner sounds were used. The purpose of this step was to acclimate the participant to the feeling of laying down with the mock head coil in the scanner. Specific feedback based on direct observation of the participant was given by research staff for head and/or body movements. (“You’re moving your head right now—remember, our pictures are blurry if you do this.”) Positive feedback was given as appropriate. (“You’re doing such a good job staying still!”)

The third step consisted of testing how well the participant did in the mock scanner without feedback for three minutes. The purpose was to determine how much the participant moved at baseline without training. MoTrak software (at this point only visible to research staff) was used to record the amount of participant head movement. No scanner sounds were used in this step. After three minutes, the participant was given feedback about motion throughout the scan. (“You did a good job keeping your arms still, but you moved your feet throughout the scan, and this caused your head to move. Remember, we want your entire body to stay still so we can take nice pictures of your brain.”)

In the fourth step, the participant was brought out of the scanner and an incentive was introduced. Specifically, participants were shown a collection of toys and were allowed to choose three. It was explained that if they stayed still throughout the scan, they would have a chance to win all three based on limiting motion during the rest of the procedure. The purpose of introducing the incentive was to determine how the participant responded to this motivating factor and to determine how this changed head motion compared to the baseline mock scan obtained during step three. For example, if the participant exhibited significant head movement during the mock scan in step three but then reduced head motion in response to the prizes, this was noted by the research staff, and the prize system during the actual MRI protocol (described below in the “Additional steps to limit motion” section) was adjusted accordingly, typically by allowing a participant to win two extra prizes per scanning session.

In the fifth step, the participant was put back into the mock scanner for three minutes, and head motion was tracked without sounds. If the participant moved their head fewer times than the mock scan in step three, they could win their first prize. No feedback was given by research staff during the scan; feedback about the total amount of head movement was given after the scan. (“You moved out of the green circle only two times that scan, so you win your first prize, but we noticed you still moved your feet quite a bit. Remember we want you to stay as still as a statue.”)

In the sixth step, research staff worked with the participant to show the effects of movement. For example, staff would have the participant look at the target and observe how much the cursor moved on the target in response to different movements of the participant’s hands and feet; after yawning; after taking a deep breath; after itching/fidgeting, etc. (“When you move your hands, your head also moves out of the green circle—did you see that? This is why we want your entire body to be still in the scan.”) The purpose of this step was to demonstrate what it means to lie still and to show that even though the participant might think they are laying still, they often might be moving in subtle ways that cause head movements.

In the seventh step, the participant completed a mock scan for five minutes with scanner sounds and with the MoTrak target system visible to the participant. Research staff gave specific verbal feedback to the participant throughout the scan, approximately every 20-30 seconds. (i.e. “You’re moving your hands a lot and this is causing your head to move out of the green circle. Remember we want your entire body to be as still as a statue.” “You’re doing such a good job laying still!”) The purpose of this step was to simulate a real scan through use of the scanner sounds and to train the participant to lie still. Before this mock scan, it was explained to the participant that if they stayed as still over the next three scans as they did during the fifth scan (i.e. moved out of the green circle fewer times) and if they responded to verbal feedback, they could win their second prize.

In the eighth step, the participant completed a mock scan for 4 minutes and 45 seconds while watching a movie clip with scanner sounds being played. This step was completed twice. The target system was no longer visible to the participant, though head movement was still tracked through the MoTrak system. The MoTrak system was configured so that every time the participant moved out of the green circle, the movie paused. Feedback was then given to the participant. (“Did you see that the movie stopped? This was because you were wiggling your hands and it caused your head to move. Remember to keep your hands still.”) Feedback was given between the first and second scan about the total number of times the participant moved out of the green circle.

In the ninth step, the participant completed a five-minute mock resting-state scan with scanner sounds. This step was completed twice. A fixation cross was shown to the participant in place of the MoTrak target. Head movement was still tracked through MoTrak. It was explained to the participant that no feedback would be given during the scan, that research staff would record the number of times the cursor moved out of the green circle, and that feedback would be given in between scans. It was also explained that the purpose of this step was to simulate what it would be like during a real scan (i.e. research staff would not be able to talk to the participant during the scan; there would be no target system presented during the actual scans; feedback would only be given in between scans, etc.) Finally, research staff explained that if the participant moved out of the green circle fewer times during each rest scan than they did in the second movie clip and if they responded to verbal feedback, they could win their third prize.

| **Step of Formal Mock Scan Protocol** | **Description** | **Purpose** | **Type of stimuli** | **Type of feedback** | **Time** |
| --- | --- | --- | --- | --- | --- |
| 1. Introduction to scanner | Show participant mock scanner; explain set-up and goals of session; answer any questions | Desensitize participant to mock scan environment | - | - | 3 minutes |
| 1. Participant preparation   Initial introduction to laying in scanner | Insert ear plugs; put on headphones and MoTrak headband; adjust mirror  Participant lay in mock scanner | Explain the purpose of all equipment  Desensitization | -  Scanner sound *off*  Screen *off* | -  Verbal feedback during the scan | 2-3 minutes  3 minutes |
| 1. Mock scan 1 (baseline) | Have participant lay in mock scanner | Determine participant movement at baseline | Scanner sound *off*  Screen *off* | Verbal feedback after the scan | 3 minutes |
| 1. Pick prizes | Participant is brought out of the scanner and allowed to pick three prizes | Introduce incentives for staying still | - | - | 3 minutes |
| 1. Mock scan 2   (First prize) | Have participant lay in mock scanner | Determine how participant responds to incentive; compare movement to scan in step 3 | Scanner sound *off*  Screen *off* | Verbal feedback after the scan | 3 minutes |
| 1. Interactive training | Show participant how moving hands, feet, legs, etc. causes the MoTrak cursor to move | Teach participant that movement of arms, legs causes slight head movements | Scanner sound *on*  MoTrack system *on screen* | Visual (target) and verbal feedback during the scan | 3 minutes |
| 1. Mock scan 3   (Second prize) | Have participant lie as still as possible | Train participant to lie still; utilize visual and verbal feedback | Scanner sound *on*  MoTrack system *on screen* | Visual (target) and verbal feedback during the scan | 5 minutes |
| 1. Mock scan 4 | Have participant lie as still as possible | Train participant to lie still during movies and without the target feedback system | Scanner sound *on*  Movie *on screen* | Visual (movie stopped) and verbal feedback during the scan | 4 minutes 45 seconds, repeated twice (9 minutes 30 seconds total) |
| 1. Mock scan 5 (Third prize) | Have participant lie as still as possible | Train participant to lie still in the resting-state scan without the target feedback system | Scanner sound *on*    Fixation cross *on screen* | No feedback given | 5 minutes, repeated twice (10 minutes total) |

Supplemental Table 1. The steps used in the mock scan protocol.

| **Condition** | **Hedge’s *g*** | **Effect size** |
| --- | --- | --- |
| Movies: No speech, no eye contact | 1.26 | Large |
| Movies: No speech, eye contact | 1.98 | Large |
| Movies: Speech, no eye contact | 1.18 | Large |
| Movies: Speech, eye contact | 1.16 | Large |

Supplemental Table 2. The effect size of the difference between the average mean FFD value of the informal and formal mock scan groups when the movie data are grouped by clip condition. See the Supplemental Methods for a full description of each clip.


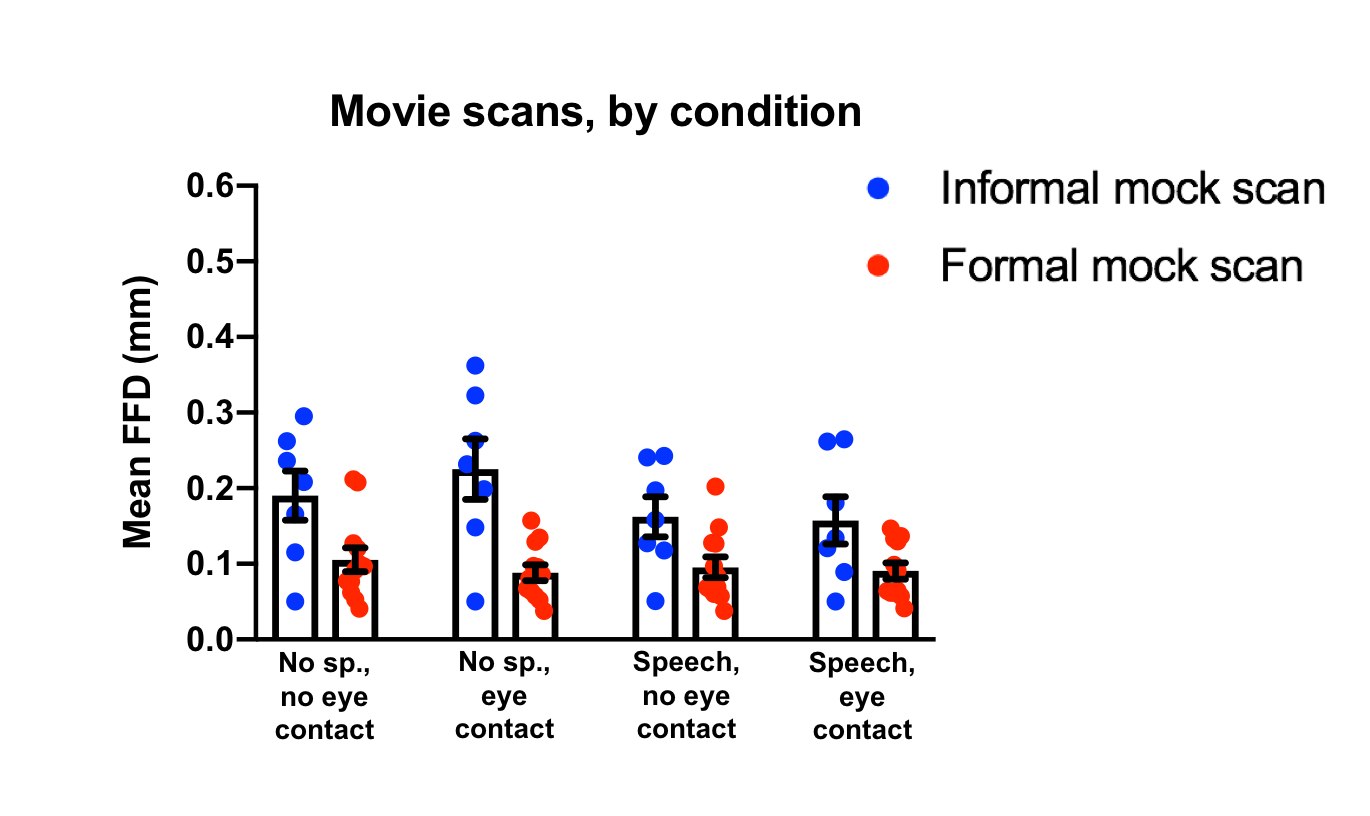


Supplemental Figure 3. Plotting the same data as in Fig. 2b, except the mean FFD over each clip is shown. Of the four conditions listed under the x-axis, each is shown twice over the four runs for a total of eight clips. See the Supplemental Methods for a full description of each clip. Figure convention as in Fig. 2 in the main text (FFD = frame-to-frame displacement; mm = millimeters; sp. = speech).

| **Test** | **Groups (males only)** | **Hedge’s *g*** | **Effect size** |
| --- | --- | --- | --- |
| Effect size: difference in grand mean FFD | Informal mock scan group vs. formal mock scan group | 2.38 | Large |
|  | Informal mock scan group vs. replication group | 2.38 | Large |

Supplemental Table 3. Effect size of the difference in grand mean FFD in mm (average mean FFD over all functional scans for a participant) among the males of each group. The direction of the effect in both cases is such that the informal mock scan group has a higher grand mean FFD.

Description of informal mock scan protocol

Participants in the informal mock scan group received a less intensive mock scan. After a 2-3 minute period of desensitization (i.e. laying on the mock scan table outside of the mock bore), participants simply lay in the mock scanner for 5-10 minutes while gradient sounds from the scanner were played (the exact time depended on their comfort level—if participants said they felt comfortable after 5 minutes, the session was concluded). Participants were given feedback for gross head movements or other large movements. No other specific feedback was given. The corresponding author (CH) conducted these scans, along with SF and KJ.

Image acquisition parameters

All subjects were scanned on a 3 T Siemens Prisma system at the Yale Magnetic Resonance Research center. We acquired a high-resolution T1-weighted 3D anatomical scan using a magnetization prepared rapid gradient echo (MPRAGE) sequence with the following image parameters: 208 contiguous slices acquired in the sagittal plane, repetition time (TR) ​= ​2400 ​ms, echo time (TE) ​= ​1.22 ​ms, flip angle ​= ​8°, slice thickness ​= ​1 ​mm, in-plane resolution ​= ​1 ​mm ​× ​1 ​mm, matrix size ​= ​256 ​× ​256. A T1-weighted 2D anatomical scan was acquired using a fast low angle shot (FLASH) sequence with the following image parameters: 75 contiguous slices acquired in the axial-oblique plane parallel to AC-PC line, TR ​= ​440 ​ms, TE ​= ​2.61 ​ms, flip angle ​= ​70°, slice thickness ​= ​2 ​mm, in-plane resolution ​= ​0.9 ​mm ​× ​0.9 ​mm, matrix size ​= ​256 ​× ​256. A T2-weighted 3D fast spin echo image was acquired using a sampling perfection with application optimized contrasts using different flip angle evolution (SPACE) sequence with the following image parameters: 208 slices per slab acquired in the sagittal plane, TR = 3200 ms, TE = 316 ms, slice thickness = 1 mm, in-plane resolution = 1 mm x 1 mm, matrix size = 256 x 256 x 208.

Functional images were acquired using a multiband gradient echo-planar imaging (EPI) pulse sequence with the following image parameters: 75 contiguous slices acquired in the axial-oblique plane parallel to AC-PC line, TR = 1000 ms, TE 30 ms, voxel size = 2.0 mm^3^, flip angle = 55 degrees, slice thickness = 2 mm, bandwidth = 1894 Hz/pixel, matrix size = 110 x 110, field of view = 220 mm, multiband factor = 5.

Description of functional runs

All tasks were presented using Psychtoolbox (version: 3.0.14; <http://psychtoolbox.org/>; MATLAB version R2018a) on a Lenovo IdeaPad 720S computer, with Ubuntu 16.04 LTS installed.

*gradCPT*

gradCPT is a continuous attention task that has been described in detail elsewhere^1-3^. Briefly, participants viewed grayscale images of city and mountain scenes presented at the center of the screen. In each trial, an image transitioned from one to the next through linear pixel-by-pixel interpolation. Each transition took 1000 ms. For 1000 ms the current scene transitioned from the previous scene, and for the next 1000 ms it transitioned to the next. Subjects were told to respond by pressing a button for city scenes and to withhold button presses for mountain scenes. City scenes occurred randomly 90% of the time. As in previous uses of gradCPT, accuracy was emphasized without reference to speed. All participants practiced outside of the scanner for 30 seconds to gain familiarity with the task.

*Movies*

The movie runs utilized a novel version of a free-viewing Selective Social Attention task^4, 5^, in which an actress is situated at the center of the screen and is surrounded by four toys in corners of the screen (Supplemental Fig. 4). Four conditions were used: The first clip was a direct gaze condition with speech, in which the actress spoke in full sentences (e.g. “Have you ever seen a monkey? Monkeys eat bananas, swing in trees, and chase each other.”) and used child- and adolescent-friendly language while smiling and making eye-contact with the camera. The second clip was a direct gaze condition with no speech, in which the actress smiled directly at the viewer while not speaking. The third clip consisted of the actress looking down at the table while speaking in full sentences and smiling (i.e. this is similar to the first clip except no eye contact was made with the viewer). The fourth clip consisted of the actress looking down at the table while smiling and not speaking. Each clip lasted two minutes and was shown twice over four runs, such that eight clips were shown total. In between clips during each run, a white fixation cross on a black background was shown for 15 seconds. Clip order was counterbalanced across participants.


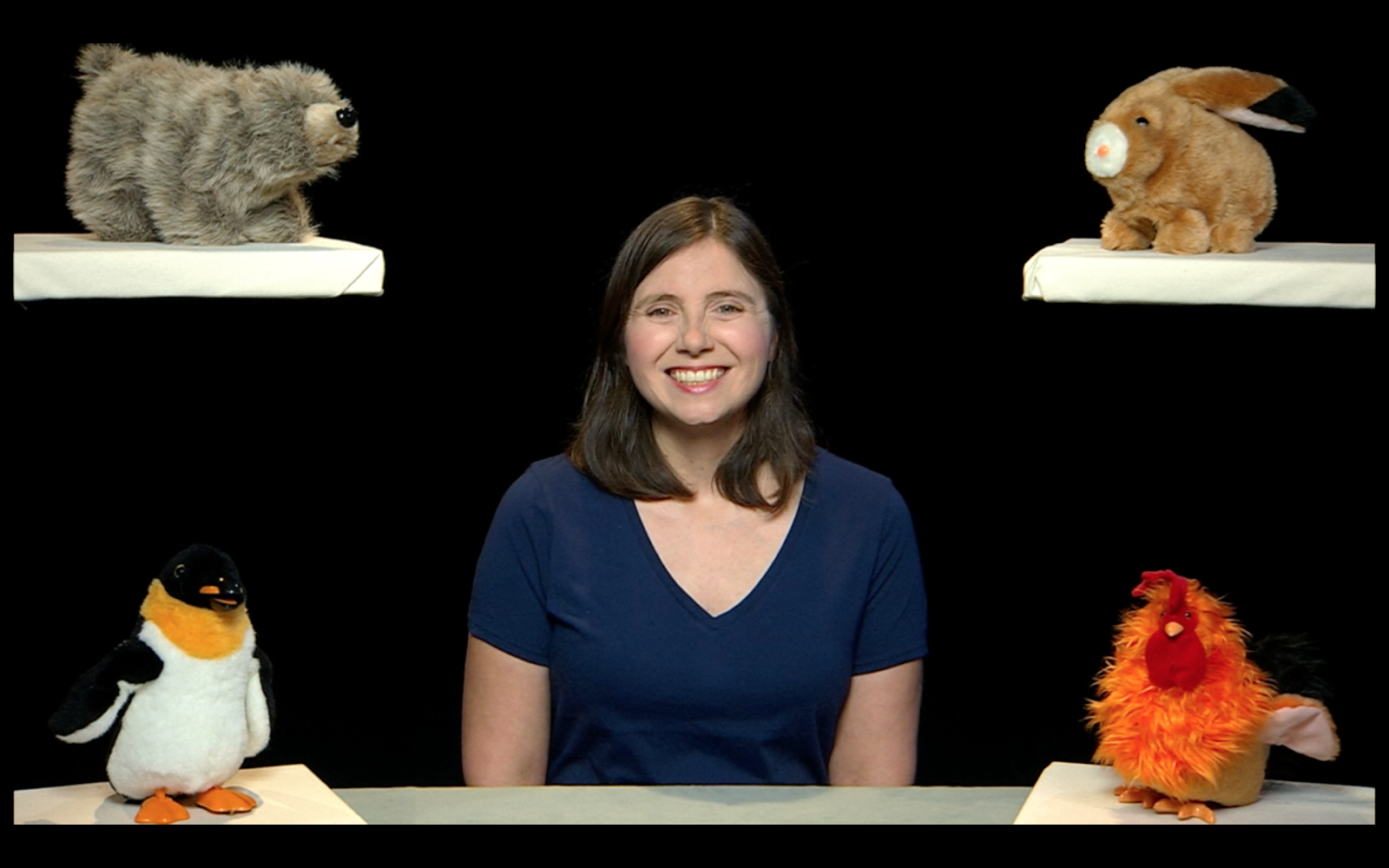


Supplemental Figure 4. Single frame from the direct gaze with speech condition of the selective social attention task. Four conditions were used: direct gaze with speech, direct gaze with no speech, no direct gaze with speech, and no direct gaze with no speech.

*Resting-state*

Subjects were instructed to keep their eyes open, relax, and think of nothing in particular while they viewed a white fixation cross on a black screen.

Additional details from MRI session

For all subjects if a participant exhibited a gross head movement, was not complying with task instructions, etc., we repeated the appropriate scan (since the goal of the ongoing study is to obtain as much high-quality data as possible) if time permitted. For this study, we had to repeat a scan three times: one subject from the informal mock scan group (subject 6, movie 4; mean FFD of first scan = 0.3323 mm; mean FFD of repeat scan = 0.4433 mm); one from the formal mock scan group (subject 18, movie 3; mean FFD of first scan = 0.2969 mm; mean FFD of repeat scan = 0.2167 mm); and one from the replication group (subject 4, movie 3; mean FFD of first scan = 0.2007 mm; mean FFD of repeat scan = 0.1985 mm). For each subject, we used the scan with lower mean FFD for the analyses described here.

| **Scan** | **Duration** |
| --- | --- |
| 1. Localizer | 10 secs |
| 1. MPRAGE | 5 mins 45 secs |
| 1. T1-FLASH | 2 mins 39 secs |
| 1. gradCPT run 1 | 5 mins 26 secs |
| 1. gradCPT run 2 | 5 mins 26 secs |
| 1. T2-SPACE | 11 mins 7 secs |
| 1. Movie run 1 | 4 mins 57 secs |
| 1. Movie run 2 | 4 mins 57 secs |
| 1. Movie run 3 | 4 mins 57 secs |
| 1. Movie run 4 | 4 mins 57 secs |
| 1. Resting-state run 1 | 5 mins 26 secs |
| 1. Resting-state run 2 | 5 mins 26 secs |
| Total scan time | 61 mins 13 secs |
| Total time spent in magnet with eye-tracker setup, time between scans, etc. (approximate) | 80-90 mins |

Supplemental Table 4. The scans conducted in this study. Note that the scans are listed in the order they are acquired. The scan times for all functional runs include shimming and magnet equilibration.

**Supplemental References**

1. Esterman, M., Noonan, S.K., Rosenberg, M. & Degutis, J. In the zone or zoning out? Tracking behavioral and neural fluctuations during sustained attention. *Cereb Cortex* **23**, 2712-2723 (2013).

2. Rosenberg, M., Noonan, S., DeGutis, J. & Esterman, M. Sustaining visual attention in the face of distraction: a novel gradual-onset continuous performance task. *Atten Percept Psychophys* **75**, 426-439 (2013).

3. Rosenberg, M.D.*, et al.* A neuromarker of sustained attention from whole-brain functional connectivity. *Nat Neurosci* **19**, 165-171 (2016).

4. Chawarska, K., Macari, S. & Shic, F. Context modulates attention to social scenes in toddlers with autism. *J Child Psychol Psychiatry* **53**, 903-913 (2012).

5. Shic, F., Wang, Q., Macari, S.L. & Chawarska, K. The role of limited salience of speech in selective attention to faces in toddlers with autism spectrum disorders. *J Child Psychol Psychiatry*  (2019).
